# Pathogenic Factors Correlate With Antimicrobial Resistance Among Clinical *Proteus mirabilis* Strains

**DOI:** 10.3389/fmicb.2020.579389

**Published:** 2020-11-25

**Authors:** Aneta Filipiak, Magdalena Chrapek, Elżbieta Literacka, Monika Wawszczak, Stanisław Głuszek, Michał Majchrzak, Grzegorz Wróbel, Małgorzata Łysek-Gładysińska, Marek Gniadkowski, Wioletta Adamus-Białek

**Affiliations:** ^1^Department of Surgical Medicine with the Laboratory of Medical Genetics, Collegium Medicum, Jan Kochanowski University, Kielce, Poland; ^2^Department of Mathematics, Jan Kochanowski University, Kielce, Poland; ^3^National Medicines Institute, Warsaw, Poland; ^4^Department of Anatomy, Collegium Medicum, Jan Kochanowski University, Kielce, Poland; ^5^Division of Medical Biology, Institute of Biology, Jan Kochanowski University, Kielce, Poland

**Keywords:** pathogenic factors, antimicrobial resistance, *P. mirabilis*, swarming motility, biofilm

## Abstract

*Proteus mirabilis* is the third most common etiological factor of urinary tract infection. It produces urease, which contributes to the formation of a crystalline biofilm, considered to be one of the most important virulence factors of *P. mirabilis* strains, along with their ability to swarm on a solid surface. The aim of this study was to analyze the pathogenic properties of two selected groups of clinical *P. mirabilis* isolates, antimicrobial susceptible and multidrug resistant (MDR), collected from hospitals in different regions in Poland. The strains were examined based on virulence gene profiles, urease and hemolysin production, biofilm formation, and swarming properties. Additionally, the strains were characterized based on the Dienes test and antibiotic susceptibility patterns. It turned out that the MDR strains exhibited kinship more often than the susceptible ones. The strains which were able to form a stronger biofilm had broader antimicrobial resistance profiles. It was also found that the strongest swarming motility correlated with susceptibility to most antibiotics. The correlations described in this work encourage further investigation of the mechanisms of pathogenicity of *P. mirabilis*.

## Introduction

Following *Escherichia coli* and *Klebsiella pneumoniae*, *Proteus mirabilis* is the third most common etiological factor of urinary tract infection (UTI) (Różalski et al., 2012; Cestari et al., 2013; Kwiecinska-Pirog et al., 2016; Peng et al., 2016; Armbruster et al., 2018), being mainly responsible for complicated UTIs or UTIs in long-term catheterized patients. Furthermore, the ability of *P. mirabilis* to form apatite or/and struvite stones in the bladder and the kidney causes severe pain in patients and augments therapeutic difficulties (Wei et al., 2014; Kwiecinska-Pirog et al., 2016; Norsworthy and Pearson, 2017). Besides UTIs, this pathogen develops diverse diseases of the respiratory tract and infections of the skin and soft tissue (including postoperative wounds, burns, etc.) (Fernández-Delgado et al., 2015). *P. mirabilis* expresses several virulence factors that allow for, e.g., effective motility against the stream of urine, uptake of nutrients, or protection from the host defense system. The most typical are fimbriae, which in general mediate attachment to uroepithelial cells. *P. mirabilis* carries genes of 17 distinct fimbrial structures, the most important being mannose-resistant *Proteus*-like pili (MR/P), *P. mirabilis* P-like pili (PMP), *P. mirabilis* fimbriae (PMF), ambient-temperature fimbriae (ATF), and uroepithelial cell adhesin (UCA) (Różalski et al., 2012; Fernández-Delgado et al., 2015). Other significant virulence factors include toxins (HpmAB), iron and zinc uptake systems, proteases and flagella (Różalski et al., 1997, 2012; Cestari et al., 2013; Armbruster et al., 2018), and urease which hydrolyses urea to ammonia and carbon dioxide. This activity is a substantial source of nitrogen for bacteria and also contributes to the formation of crystalline biofilm that blocks the catheter lumen which is considered as one of the most important virulence factors of *P. mirabilis* (Pearson et al., 2011; Armbruster and Mobley, 2012; Czerwonka et al., 2016; Kwiecinska-Pirog et al., 2016). The pathogenicity of *P. mirabilis* strains could also be associated with their ability to swarm on a solid surface (Norsworthy and Pearson, 2017). This phenomenon relies on the conversion of short swimmer cells into long, hyper-flagellated swarmer cells and has been used in a simple Dienes test to differentiate *Proteus* spp. isolates. Its principle is based on the occurrence of boundaries between zones of the swarming growth of non-related strains, while those produced by isogenic strains fuse with each other. However, the background of this effect remains unclear (Drzewiecka, 2016).

Over the past decades, clinical strains of *P. mirabilis*, just like other Enterobacterales, have become increasingly resistant to antimicrobials, which is a serious problem for hospitalized patients (Baraniak et al., 2016; Köck et al., 2018; Literacka et al., 2019; Mirzaei et al., 2019). In some countries, strains with extended-spectrum β-lactamases (ESBLs) or AmpC-like cephalosporinases have spread, which, apart from resistance to penicillins and cephalosporins (including oxyimino-compounds), display broad resistance to other anti-infectives (D’Andrea et al., 2011; Izdebski et al., 2013; Adler et al., 2016; Brolund et al., 2019; Literacka et al., 2019). In Poland, CMY-2-like AmpC producers may account for more than 20% of *P. mirabilis* isolates causing nosocomial infections (Empel et al., 2008). The aim of this study was to check how antibiotic resistance generally correlates with the virulence of clinical *P. mirabilis* strains.

## Materials and Methods

### Bacterial Strains and Antimicrobial Susceptibility Testing

Fifty non-duplicate clinical *P. mirabilis* isolates deposited in the National Medicines Institute in Warsaw, Poland, were used in this study. They were recovered from the urine of patients treated in 24 hospitals in 18 Polish cities from 1998 to 2004. The strains were stored in 15% glycerol stocks at −80°C. Each analysis was performed on a fresh bacterial culture. Apart from the broad geographic distribution, the isolates varied in antimicrobial susceptibility. Generally, the two distinct groups of bacterial strains were used—25 multidrug-resistant (MDR) strains and 25 fully sensitive bacterial strains. The MDR *P. mirabilis* strains included 15 isolates with CMY-2-like AmpC cephalosporinases (CMY-12, -14, -15, and -45) plus TEM-1/-2-like β-lactamases, reported previously (Literacka et al., 2004; Empel et al., 2008; D’Andrea et al., 2011). The remaining isolates were selected based on their β-lactam susceptibility phenotypes, β-lactamase isoelectric focusing patterns, and β-lactamase gene PCR profiles, determined as described earlier (Literacka et al., 2004; Empel et al., 2008). These comprised eight isolates resistant to oxyimino-cephalosporins, including seven further CMY-2-like AmpC and one CTX-M-1-like ESBL producers (all with TEM-1/-2), two ampicillin-resistant isolates with TEM-1-like enzymes. These 25 β-lactamase producers were also resistant to aminoglycosides, fluoroquinolones, co-trimoxazole, and/or chloramphenicol. The minimal inhibitory concentrations (MICs) of 19 antimicrobials (Supplementary Table S1) were evaluated by broth microdilution. The methodology and results interpretation were in accordance with the European Committee on Antimicrobial Susceptibility Testing guidelines, 2019^1^, and, in case of cefoxitin, with the Clinical Laboratory Standards Institute (Clinical and Laboratory Standards Institute, 2019).

### Virulence Gene Detection

Bacterial DNA was isolated and purified with the GenEluteTM Bacterial Genomic DNA kit (Sigma-Aldrich, St. Louis, Missouri, United States). PCR was used for the identification of six virulence factor genes (*fliL*, *mrpA*, *pmfA*, *ureC*, *zapA*, *hpmA*, *hpmB*, and *uca*). The PCRs were performed in 25 μl reaction mixtures containing 12.5μl of DreamTaq^TM^ Green DNA Polymerase Master Mix (2×) (Thermo Fisher Scientific, Waltham, MA, United States), bacterial DNA (1 ng), and 100 pmol of each primer (Institute of Biochemistry and Biophysics, Polish Academy of Sciences, Warsaw, Poland)^2^. The sequences of primers, their annealing temperatures, and their amplicon sizes are shown in Table 1. The cycling conditions were as follows: denaturation at 94°C for 2 min, followed by 30 cycles of 1 min at 94°C, 1 min at varying annealing temperature, and 1 min at 72°C, followed by 5 min at 72°C (Mastercykler^®^ Nexus, Eppendorf, Juelich, Germany).

**TABLE 1:** Oligonucleotides used for PCR.

**Gene**	**Primer**	**Sequence (5′–3′)**	**Annealing temperature (°C)**	**PCR product (bp)**	**References**
*FliL*	FliL1	CTCTGCTCGTGGTGGTGTCG	57	770	Barbour et al., 2012
	FliL2	GCGTCGTCACCTGATGTGTC			
*mrpA*	mrpA1	ACACCTGCCCATATGGAAGATACTGGTACA	40	550	Barbour et al., 2012
	mrpA2	AAGTGATGAAGCTTAGTGATGGTGATGGTGATGAGAGTAAGTCACC			
*pmfA*	pmfA1	TCAGCATGTGGATTAGCAGCA	59	385	This study
	pmfA2	CTTGGAATTCACCTGGCGTT			
*hpmA*	hpmA1	GTTGAGGGGCGTTATCAAGAGTC	55	709	Cestari et al., 2013
	hpmA2	GATAACTGTTTTGCCCTTTTGTGC			
*hpmB*	hpmB1	CAGTGGATTAAGCGCAAATG	55	422	Cestari et al., 2013
	hpmB2	CCTTCAATACGTTCAACAAACC			
*Uca*	uca1	GCTGGCTCATCTATGGCGTA	60	453	This study
	uca2	AGCGGTAGATTGTCCGGTTG			
*ureC*	ureC1	TGGCAAGGCAGGTAATCCAG	58	589	This study
	ureC2	ATTGGGCTCTCCTACCGACT			
*zapA*	zapA1	TGGCGCAAATACGACTACCA	57	332	This study
	zapA2	TATCGTCTCCTTCGCCTCCA			

### Hemolysin and Urease Production

The β-hemolysis properties of *P. mirabilis* strains were determined by observing clear zones around bacterial colonies on blood agar supplemented with 5% (v/v) bovine blood (Oxoid, Basingstoke, United Kingdom) after 24 or 48 h of incubation in 37°C (Kang et al., 2019).

The ureolytic activity in Christensen broth was analyzed according to the method described previously (Cullen et al., 2015), with some modifications. A fresh bacterial inoculum (0.5 McFarland) was diluted 1:100 in Christensen broth and incubated with shaking (200 rpm) at 37°C for 6 h. After each hour of incubation, the absorbance of the centrifuged supernatant was measured spectrophotometrically at 560 nm. Additionally, the ureolytic activity was observed on Christensen agar. Briefly, 10 μl of fresh bacterial inoculum (0.5 McFarland) was dropped on the center of an agar plate. The culture was incubated at 37°C for 7 h, and the medium color change (from yellow to pink) was indicative of urea hydrolysis. The diameter of a pink color zone around the culture was measured after each hour of incubation, starting from the third hour. The dynamics of the ureolytic activity was estimated based on the percentage difference of the activity values between the previous and the next hour of incubation.

### Swarming Motility and the Dienes Test

The Dienes test was performed according to the protocol described previously (Pfaller et al., 2000), with some modifications. Ten microliters of overnight broth cultures was inoculated on Luria–Bertani agar plates. Four aliquots of individual cultures were applied onto a single plate at equal distances (about 1 cm) from each other, pre-dried for 15 min at room temperature, and incubated at 37°C for 24 h. Bacterial strains showing a clear band in between (Dienes demarcation line) were interpreted as unrelated, while those without the Dienes line were regarded as related to each other. Additionally, the expansiveness/rate of swarming motility was evaluated for each strain by its proportional coverage of the plate after overnight incubation compared to other strains cultured on the same plate. The expansiveness was classified into three categories: category 1, weak swarming (coverage < 5%); category 2, medium swarming (5– ≤ 25%); and category 3, intensive swarming (25– ≤ 50%). The bacterial behavior in each combination of strains was tested in triplicate.

### Biofilm Formation

The ability of *P. mirabilis* strains to form a biofilm was analyzed on glass and polyurethane surfaces according to the method described previously (Adamus-Białek et al., 2015). The biofilm on the glass was stained with SYTO^®^ 9 solution and propidium iodide, according to the manufacturer’s protocol (Filmtracer^TM^; Invitrogen, Carlsbad, California, United States), and then observed with an epi-fluorescence microscope (Axio Scope.A1; ZEISS, Oberkochen, Germany) on coverslips. One strain was cultured on three coverslips, and five of the most representative images were photographed. All strains were classified into three groups of the biofilm assessment scale: group 1, lack of biofilm, single cells observed; group 2, single microcolonies of biofilm; and group 3, biofilm covering the entire coverslip. Biofilm formation on the polyurethane was measured spectrophotometrically at 531 nm after the overnight incubation of bacterial strains in LB broth and crystal violet staining (Adamus-Białek et al., 2015). The biofilm was measured in triplicate of two independent experiments. The strains unable to form a biofilm were classified based on crystal violet adsorption on the polyurethane, measured as *A*_531_ < 0.08. This value corresponds with the control incubation of crystal violet without bacteria. The relative biofilm (*B*_Rel_, biofilm formation level independent of the bacterial growth rate) was estimated as the proportion between raw values of absorbance of the adsorbed crystal violet and density of bacterial growth (*A*_531_/*A*_600_).

### Statistical Analysis

The group comparisons of *P. mirabilis* strains were carried out using Fisher exact test for categorical variables, unpaired *t*-test for quantitative, normally distributed variables, or Mann–Whitney test for quantitative, non-normally distributed variables (the normality of distribution was checked with the Shapiro-Wilk test). The cluster analysis was performed using Euclidean distance and Ward’s method. The statistical tests were two-tailed, and a *p*-value less than 0.05 was considered as significant. All statistical analyses were performed using R (version 3.1.2; The R Foundation for Statistical Computing, Vienna, Austria) and Graph Pad Prism v. 6 (San Diego, CA, United States). The statistical analysis took into account standard deviations, and they are based on data obtained from repeated experiments.

## Results

### Antimicrobial Susceptibility

In general, the study sample was selected based on antibiotic susceptibility patterns, comprising a group of 25 susceptible strains and a group of 25 MDR strains, the latter one including 22 isolates with CMY-2-like AmpCs, one isolate with a CTX-M-1-like ESBL (all co-producing TEM-1/-2 β-lactamases), and two isolates with TEM-1-like enzymes only (Supplementary Table S1). The β-lactam susceptibility patterns, reported previously for 15 CMY producers (Literacka et al., 2004; D’Andrea et al., 2011), corresponded well to the β-lactamase content of the isolates, briefly with resistance to penicillins and oxyimino-cephalosporins in AmpC or ESBL producers, and to penicillins only in TEM-1 producers. Resistance to aminoglycosides, fluoroquinolones, co-trimoxazole, and/or chloramphenicol occurred frequently in the 25 β-lactamase producers, defining each of these as MDR (Magiorakos et al., 2012) despite the variety of individual susceptibility patterns and levels of resistance to particular compounds. Otherwise, all the 25 β-lactamase negatives were susceptible to all antimicrobials tested (multidrug susceptible, MDS). The lower *in vitro* activity of imipenem, characteristic of *Proteus* and related genera (see footnote), was observed among both MDR and MDS isolates, however, with higher MICs among the former ones.

### Virulence Factors

**FIGURE 1:**
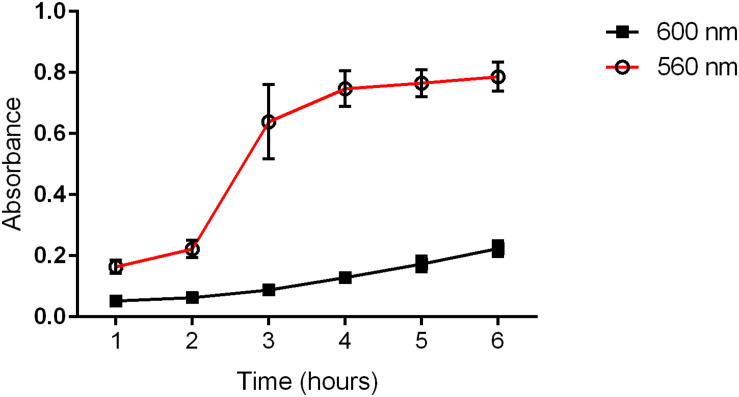
Medium ureolytic activity (560 nm) and bacterial growth (600 nm) of clinical *P. mirabilis* strains. The absorbance was measured spectrophotometrically in each subsequent hour of incubation.

All the *P. mirabilis* strains were positive for the studied pathogenicity-related genes (*fliL*, *mrpA*, *pmfA*, *ureC*, *zapA*, *hpmA*, *hpmB*, and *uca*). Eighty-four percent of the isolates produced a transparent zone or color change on blood agar; however, only eight strains (16%) exhibited the typical β-hemolytic activity. The rate of ureolytic activity was measured in a time course, and it was independent of the bacterial growth rate (Figure 1). All the strains hydrolyzed urea, but the lowest and the highest level of urease activity in the first and the last hour varied in the range of approximately 30%. The highest increase in urease activity was detected in the third hour of incubation in Christensen broth. A similar observation was done during the incubation of agar medium where the enlargement of the ureolytic zone was observed at different rates for individual strains (differences between the strains were from 72 to 58% for the 3rd to 6th hour of incubation, respectively). Generally, despite the differences between strains, the ureolytic activity was stable in the time course.

### Swarming Motility and the Dienes Test

The *P. mirabilis* strains were checked for their swarming motility rate. Twenty percent of the examined isolates exhibited weak swarming growth, 36% showed medium swarming, and 44% displayed intensive swarming. Considering the Dienes test, the strains exhibited kinship with different numbers of other isolates (Table 2). The lowest number of kinships was expressed by four strains that exhibited confluent growth with only three other strains, and the highest number was observed in the case of one strain, which was kindred with 31 others.

**TABLE 2:** Summary of the Dienes test results.

Number of strains	4	4	3	4	1	8	5	7	2	1	1	1	1	2	2	1	2	1
Number of kin strains	3	4	5	6	7	8	9	10	11	12	13	14	15	16	20	27	29	31

*The differentiation of P. mirabilis strains was prepared based on the number of strains which are kindred with other strains.*

### Biofilm Formation

The *P. mirabilis* strains were tested for their ability to form a biofilm. The absorbance values of the crystal violet adsorbed on the polyurethane surface were different for individual strains, and these ranged between 0.079 and 1, with the medium absorbance level at 0.4. Only in the case of two strains was the absorbance level consistent with the control value. We observed that the biofilm formation on glass surfaces [three groups of the biofilm assessment scale (Supplementary Figure S1)] positively correlated with the relative biofilm (*B*_Rel_) on the polyurethane: the strains unable to form a biofilm on the glass exhibited a significantly weaker biofilm on the polyurethane, compared to the strains forming a stronger biofilm (microcolonies or entire coverslip covered) [*p* < 0.05, unpaired two-tailed *t*-test (Supplementary Figure S2)]. Significantly more strains were classified as unable to form a biofilm on the glass (42%) than on the polyurethane surface (4%).

### Correlations

**FIGURE 2:**
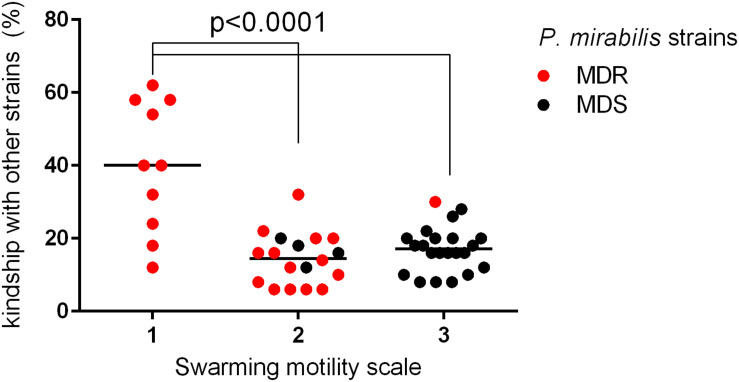
Correlation between kinship and swarming motility among sensitive and multidrug-resistant (MDR) *P. mirabilis* strains. The bacterial strains (*n* = 50) were differentiated based on different levels of swarming motility (in a scale of 1, 2, and 3) and analyzed according to kinship with other strains (% of strains kindred with a particular strain) (red dot, MDR; black dot, sensitive strain). The median value was marked with a black line. The unpaired two-tailed *t*-test was used for a statistically significant difference (*p* < 0.05) between the groups of strains, which exhibited a different scale of swarming motility (Graph Pad Prism v. 6).

A more detailed comparison of the results obtained in the above-mentioned experiments allowed us to observe several dependencies. The kinship of the examined strains correlated with their MIC value profiles (Table 3). For each strain, the number of kindred strains and the number of not kindred strains were noted. Accordingly, the MIC values of all antibiotics of that individual strain were compared to adequate MICs of these kindred or not kindred strains. That comparison was based on the percentage similarity of MIC values between a given strain and other kindred or not kindred strains with it. Forty-four percent of the strains (*n* = 22) revealed a significant correlation between kinship and susceptibility profiles; they mostly belonged to the MDS group (16 of 22). Eighty-six percent of these (19/22) had more similar antibiotic sensitivity patterns (MIC similarity) with the group of not kindred strains than with kindred strains. Only in three cases did the kin strains had similar susceptibility profiles. The kinship also correlated with the weaker swarming motility of the *P. mirabilis* strains. The strains with weaker swarming growth were kindred with a higher number of strains compared to those with stronger swarming growth (Figure 2). Additionally, kinship with a higher number of strains was observed more often among MDR strains (*p* = 0.0148, unpaired two-tailed *t*-test).

**TABLE 3:** Correlation between kindship of *P. mirabilis* strains measured by the Dienes test and median percentage similarity of their minimal inhibitory concentration (MIC) values for all the antibiotics analyzed (MICs similarity).

***P. mirabilis* strain**	**Other strains**				***p*-value**
	**Kindred**		**Not kindred**		
	**No.**	**MICs similarity (%)**	**No.**	**MICs similarity (%)**	
368	8	35	40	95	0.0386
*845*	9	80	39	50	0.0016
*2014*	9	85	39	40	0.0003
*2198*	14	50	34	70	0.0000
*3010*	28	30	10	57.5	0.0174
5112	10	37.5	38	95	0.0119
5618	7	30	41	95	0.0092
5653	9	35	39	95	0.0219
5663	12	30	36	95	0.0076
5777	6	37.5	42	95	0.0528
5778	7	30	41	95	0.0105
*6103*	15	35	25	55	0.0066
*6187*	6	85	42	30	0.0099
6365	8	32.5	40	95	0.0194
6405	7	35	41	95	0.039
6521	13	30	35	95	0.0086
6771	9	30	39	95	0.0138
7101	9	30	39	95	0.0348
7104	8	32.5	40	95	0.0473
7498	7	30	41	95	0.016
7499	5	30	43	95	0.0163
7882	9	35	39	95	0.0004

*The statistically significant (p < 0.05) differences were assessed with the use of Mann–Whitney test; only statistically significant results were included in the table. The italicized number of strains means multidrug resistant.*

**FIGURE 3:**
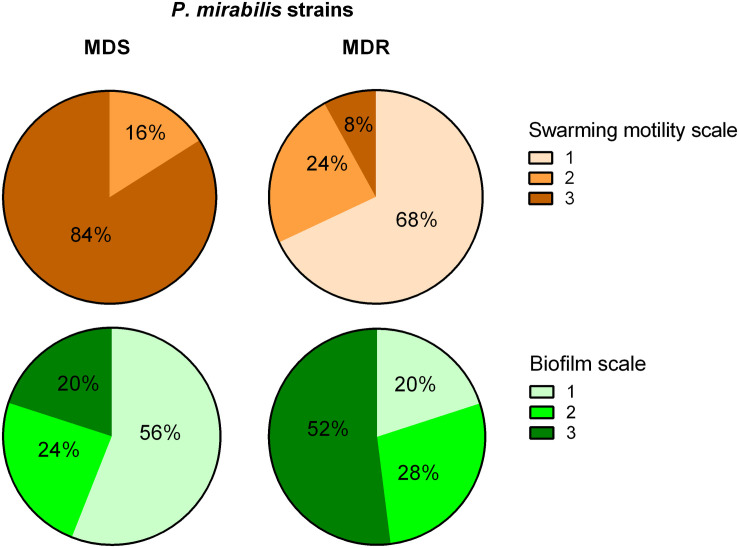
Biofilm and swarming motility among multidrug-susceptible (MDS) and multidrug-resistant (MDR) *P. mirabilis* strains. A statistically significant difference was observed between MDS and MDR *P. mirabilis* strains in terms of biofilm formation on the glass (chi-square test, *p* = 0.0161) and swarming motility (chi-square test, *p* = 0.0001) measured in a three-stage scale (Graph Pad Prism v. 6). Biofilm scale: 1, lack of biofilm, single cells observed; 2, single microcolonies of biofilm; 3, biofilm covering the entire coverslip. Swarming motility scale: 1, weak swarming (coverage < 5%); 2, medium swarming (5– ≤ 25%); 3, intensive swarming (25– ≤ 50%).

The MDR and the MDS groups of strains were compared based on expansiveness of swarming and biofilm strength (Figure 3). Eighty-four percent of susceptible strains exhibited the highest swarming motility rate, and these did not include any isolate of the weakest swarming growth. In contrast, 68% of MDR strains exhibited the weakest swarming and only 8% of them showed the strongest motility rate. Additionally, 56% of MDS strains were not able to form a biofilm, whereas 52% of MDR strains covered the entire coverslips (the strongest biofilm formation). Otherwise, the strongest biofilm was formed by only 20% of MDS strains, and the lack of biofilm was observed in only 20% of the MDRs. Similar observations were made in the case of biofilm formation on the polyurethane: the susceptible isolates revealed a significantly weaker biofilm when compared to the resistant strains (unpaired Mann–Whitney test, *p* = 0.0371).

### Differentiation of the Isolates

**FIGURE 4:**
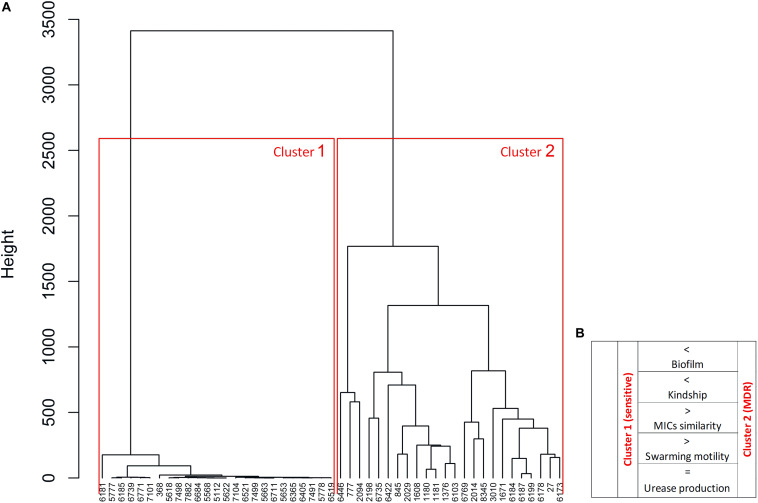
Differentiation of *P. mirabilis* strains based on the profiles of minimal inhibitory concentrations (MICs) of antibiotics. The similarities of MICs of antibiotics were used for the dendrogram **(A)** developed by Ward’s agglomeration. The schematic characteristic of cluster 1 and cluster 2 was added **(B)** based on the bacterial features, where >, < higher/stronger, = the same level.

The *P. mirabilis* isolates were differentiated using the Euclidean distance between the MIC values of all antibiotics. As expected, two distinct clusters were observed in the dendrogram (Figure 4). Cluster 1 contained a highly homogeneous group of only susceptible strains, whereas cluster 2 with only MDR strains exhibited significantly larger differences in MICs (two tailed *t*-test, unpaired, *p* < 0.0001). This clustering with a clear distinction of bacterial antibiotic sensitivity level confirmed the correlations described above, but here we proved their interdependence. Cluster 1 represents the strains kindred with fewer strains compared to cluster 2 (two tailed *t*-test, unpaired, *p* = 0.017), i.e., cluster 2 strains exhibited kinship to each other more often than those in cluster 1. Moreover, cluster 1 included strains forming a weaker biofilm, both on polyurethane (two tailed *t*-test, unpaired, *p* = 0.055) and on glass (two-tailed *t*-test, unpaired, *p* = 0.015), and exhibiting statistically significant more expansive swarming motility (two tailed *t*-test, unpaired, *p* < 0.001) compared to cluster 2. There was no difference either in the level or rate of urease and hemolysin production between the two clusters.

## Discussion

The study was focused primarily on pathogenicity factors of *P. mirabilis* isolates, recovered from hospital UTIs in different regions in Poland and selected based on their distinct susceptibility profiles. Previous reports have documented a remarkable epidemiological success of the MDR AmpC-producing strains in Greece, Italy, and Poland, where they accounted for approximately 20% of nosocomial clinical *P. mirabilis* isolates (Literacka et al., 2004; D’Andrea et al., 2011). This prompted us to check for other factors, including virulence properties, that might have contributed to their effective dissemination in hospital environments. We also wanted to refer to our previous studies on uropathogenic *E. coli* strains and compare these two species in some aspects of their pathogenicity.

Owing to specific virulence factors, *P. mirabilis* is particularly troublesome for catheterized patients and is responsible for complicated UTI with the development of urinary stones (Jacobsen and Shirtliff, 2011; Niveditha et al., 2012; Flores-Mireles et al., 2015). In our study, we analyzed the presence of typical virulence factor genes, starting with *fliL*, *zapA*, *hpmA*, *hpmB*, and *ureC.* These are engaged in immune system evasion and/or iron acquisition (Flores-Mireles et al., 2015); moreover, *fliL* and *zapA* are involved in swarmer cell differentiation and swarming behavior (Cusick et al., 2011; Fusco et al., 2017). Other genes included those determining the mannose-resistant *Proteus*-like adhesion (*mrpA*), *P. mirabilis* fimbriae (*pmfA*), and uroepithelial cell adhesion (*uca*), playing a crucial role in catheter-associated biofilm formation and urinary tract colonization (Nielubowicz and Mobley, 2010; Jacobsen and Shirtliff, 2011; Armbruster and Mobley, 2012). All the examined *P. mirabilis* strains carried all the virulence genes addressed, which is consistent with other reports (Sosa et al., 2006; Armbruster et al., 2018) but is in opposition to our previous observation concerning virulence genes in uropathogenic *E. coli* (Adamus-Białek et al., 2019). It might be associated with the different pathogenicity mechanisms of these two related species, where the pathogenicity of *P. mirabilis* is based rather on the variable expression of these genes and not their mere presence. The *P. mirabilis* chromosome is strongly conservative, and the location of the analyzed genes seems to be more stable (Armbruster et al., 2018) compared to *E. coli*. We also did not detect any correlation between the urease and hemolysin expression levels and the other pathogenic properties or susceptibility profiles. The ureolytic activity peaked quickly during culture incubation and was independent of the type and the rate of bacterial growth being so similar in both swimmer (in broth) and swarmer cells (on agar). This activity is critical in the formation of bacteria-induced stones and forming crystalline biofilms, which is particularly dangerous for long-term catheterized patients (Konieczna et al., 2012; Armbruster et al., 2018; Flores-Mireles et al., 2015); however, we did not observe any correlation between enhanced urease expression and a stronger biofilm.

**FIGURE 5:**
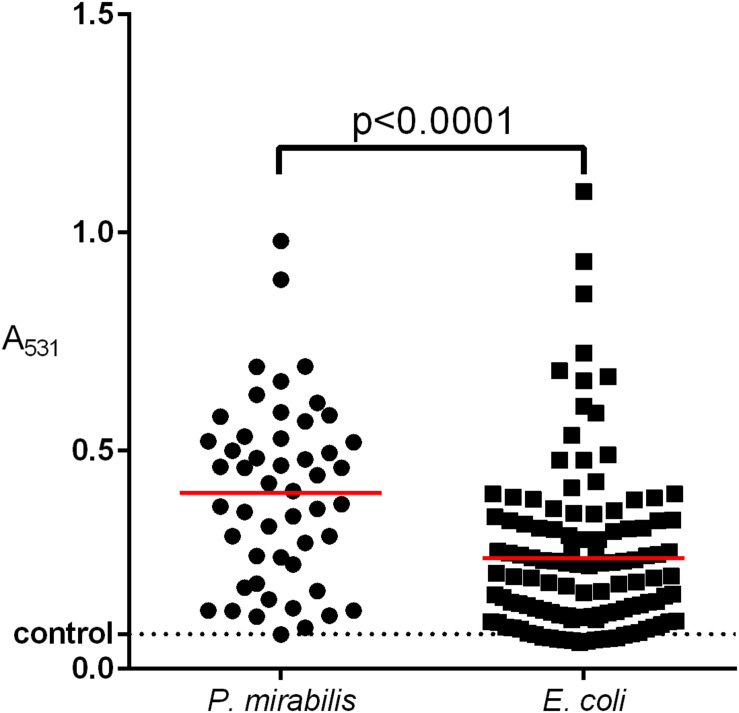
Comparison of biofilm formation between clinical *E. coli* and *P. mirabilis* strains isolated from the urine of hospitalized patients. The biofilm was measured in triplicate of two independent experiments by crystal violet absorbance at 531 nm. The median value was marked with a red line. Unpaired two-tailed *t*-test was used for a statistically significant difference (*p* < 0.05) (Graph Pad Prism v. 6).

The ability to form a biofilm varies remarkably, even among strains of the same species (Donlan, 2002; Berlanga and Guerrero, 2016; Visick et al., 2016). Our results (Figure 5) indicate that the *P. mirabilis* biofilm may be stronger than that of *E. coli* (Adamus-Białek et al., 2015). We observed significant differences of “raw” biofilm formation (*A*_531_) between *P. mirabilis* and *E. coli*, but when we considered the *B*_Rel_, the differences disappeared. That may mean that *P. mirabilis* grows faster, providing greater biofilm yield, which might be important during host invasion. The decrease of the *P. mirabilis* growth rate might inhibit biofilm formation and decrease the resistance to antibiotics. We also observed that the *B*_Rel_ formation on the glass correlated proportionally with that on the polyurethane, which was again in contrast to that of *E. coli* (Adamus-Białek et al., 2015). Czerwonka et al. (2016) reported that the high hydrophobicity of the *P. mirabilis* cell surface correlated with a low biofilm amount, which is important for hydrophobic surfaces like glass. It was proved that hydrophilic catheters may prevent catheter-associated UTIs (Rognoni and Tarricone, 2017), so it would be advisable to evaluate hydrophobicity rates that are characteristic for the majority of *P. mirabilis* clinical strains. We also showed that a stronger biofilm was formed by MDR isolates compared to the susceptible ones, which has been well evidenced and assigned to the extracellular matrix and a weaker penetration of the compounds into the bacterial community (Del Pozo, 2018; Roy et al., 2018). However, some antibiotics penetrate efficiently through biofilm (Hall and Mah, 2017), so it has been suggested that some components of the biofilm matrix may affect the activity of antibiotics (Billings et al., 2013), the accumulation of bacterial cells can amplify the activity of β-lactamases and other resistance mechanisms, and the downturn of the cellular metabolism inside the biofilm may contribute to resistance to antibiotics (Anderl et al., 2003). This persister phenomenon of dormant cells arising within bacterial biofilms with high tolerance to antibiotics may cause a relapse of infection (Lewis, 2001, 2010). Further research in this direction may yield interesting results on the biofilm activity of *P. mirabilis*.

The statistical analysis allowed us to observe specific correlations between virulence-associated properties and antimicrobial susceptibility profiles. We found that stronger swarming motility was significant among susceptible strains and *vice versa*. It was not confirmed in the case of tazobactam, the effect of which is independent of swarming motility (Nomura et al., 1997). Similar tests were conducted by Auer et al. (2018) who tested swarmer cells for sensitivity to wall-modifying antibiotics. They found that the thickness of peptidoglycan makes swarmer *P. mirabilis* cells more sensitive to osmotic pressure and thus the cell wall-targeting antibiotics. Considering that swarming is an intrinsic feature of *P. mirabilis*, the acquisition of certain antibiotic resistance mechanisms by its individual strains may affect their growth properties, including the ability to transform from swimmer to swarmer cells. New evidence about this phenomenon has been recently provided by Tipping and Gibbs (2019). This bacterial behavior is under the pressure of Ids system which mediates in territorial exclusion among *P. mirabilis* strains. The IdsE and IdsD proteins are signaling molecules which recognize themselves as matching variants among kindred strains. Disruption of this phenomenon leads to the creation of a demarcation line and inhibition of the mutual growth of neighbored strains or decreased expansion of one of them with pronounced swarming growth of the more aggressive strain. They also observed that mutants without the expression of *IdsE* exhibited increased tolerance to ampicillin and kanamycin, which is consistent with our observation that strains with weaker swarming growth had decreased tolerance to antibiotics and *vice versa*. This recognition signals also need flagellar regulation. However, despite the presence of *fliL* and *zapA* among all studied strains, we observed poor or even lack of typical swarming growth in approximately 20% of the isolates. That might be due to the low expression of these genes and/or correspondence to the significant role of other genes in this type of motility (Belas, 1994; Belas et al., 1995; Furness et al., 1997; Dufour et al., 1998; Morgenstein et al., 2010; Fusco et al., 2017; Little et al., 2018). The appearance of the demarcation line between two bacterial isolates causes a competition between them and is considered as a lack of kinship between neighboring bacterial strains (Pfaller et al., 2000; Munson et al., 2002; Drzewiecka et al., 2010; Siwińska et al., 2019). We noticed that the strains with more expansive swarming were related to less of the other strains in the Dienes test. The demarcation line is still rather poorly understood and probably regulated by multiple mechanisms, like the type VI secretion system, operons *ids*ABCDEF and *idr*ABCDE, and the hpc-vgrG effector (Alteri et al., 2013, 2017; Wenren et al., 2013). However, factors responsible for the disability of strains to extensively swarm in the place of mutual cohabitation with other strains have not been unambiguously identified. Budding et al. (2009) suggested that this characteristic might reflect environmental competition between *P. mirabilis* strains which might also have implications in hospital settings. The presented observation and other reports encourage further research into this still poorly explored phenomenon.

The Ward’s agglomeration test applied for the analyzed MIC values allowed us a holistic view on the obtained results. As expected, the isolates were separated into two clusters of MDS isolates and MDR ones. Antibiotic resistance correlated with the pathogenic properties of *P. mirabilis* differently than in the case of *E. coli* (Adamus-Bialek et al., 2009). A similar study of *P. mirabilis* was reported by Stankowska et al. (2008) who differentiated the swarming growth rate and ureotylic, proteolytic, and hemolytic activities, calculating the relative virulence index based on the cumulative scores for these activities. However, our discovered correlations indicate that virulence properties may be alternatively expressed by *P. mirabilis* strains. Biofilm and swarming growth seemed to be antagonistic to each other, ureolytic activity was similar in all the isolates, and hemolysin was rarely detected, suggesting a lower relevance of the relative virulence index. We also excluded the potential rule that kindred strains which do not form a demarcation line will have a similar drug resistance profile. The further discussion indicates the need to rethink the applied concept of kinship in relation to the demarcation line, especially that Tipping and Gibbs (2019) emphasize the complex regulation of gene expression involved in this phenomenon. We showed that kindred or rather compatible strains were less similar in terms of MIC values, expressing also broader resistance and stronger biofilm formation but less ability to swarm. This compatibility with a larger number of strains might promote mixed colonization or infection and thus increase horizontal gene transfer, producing better-adapted organisms that are harder to eradicate (Anderl et al., 2003; Billings et al., 2013). Otherwise, the strains which exhibited frequent incompatibility with others also revealed broad susceptibility to antibiotics and stronger swarming motility. These might represent a wild-type group of strains of low or no pre-exposure to antimicrobials. β-Lactams, belonging to the first-line drugs for UTI treatment, might select resistant organisms, including MDR strains with resistance also to other antimicrobial classes. That would explain their clustering in the dendrogram. Antibiotics can stimulate the mechanisms of adaptation to an unfavorable condition. In the previous study, we observed how the antibiotics changed the genetic virulence profiles of uropathogenic *E. coli* strains (Adamus-Białek et al., 2019). In the case of *P. mirabilis*, a similar phenomenon is also possible but probably in another aspect like communication and compromises between *P. mirabilis* strains.

To sum up, multidrug-resistant *P. mirabilis* strains more often exhibited mutual growth between each other, weaker swarming motility, and stronger biofilm in comparison with sensitive strains, which represented mostly incompatible growth with aggressive swarming motility and weaker biofilm. We also observed that ureolytic activity was independent of the cell type and the rate of bacterial growth, which is especially dangerous for long-term catheterized patients. Additionally, bacterial kindship identified *via* the Dienes test did not correlate with the similarity of their MIC values, which indicates diverse adaptation routes and kindship meaning. Here, for the first time, we described the specific correlations between the pathogenic properties of *P. mirabilis* strains, which encourage further investigation of swarming growth, territoriality mechanism, and other pathogenic properties.

## Data Availability Statement

The original contributions presented in the study are included in the article/Supplementary Materials, further inquiries can be directed to the corresponding author/s.

## Author Contributions

AF: laboratory work (microbiological experiments), results analyzes. MC: statistical analyzes. EL: antibiotic susceptibility testing, results analyzes. MW: laboratory work (microbiological experiments). SG: financing. MM: laboratory work. MŁ-G: laboratory work. GW: laboratory work. MG: results analysis, substantive consultation, interpretation of results, improvement of the manuscript. WA-B: concept and research plan, results analysis, interpretation of results, research management, manuscript preparation. All authors contributed to the article and approved the submitted version.

## Conflict of Interest

The authors declare that the research was conducted in the absence of any commercial or financial relationships that could be construed as a potential conflict of interest.

## Footnotes

1 www.eucast.org

2 www.oligo.pl

## Supplementary Material

Supplementary Figure 1 The exemplary observation of the biofilm on the glass after staining with SYTO^®^ 9 and propidium iodide, according to the manufacturer’s protocol, with an epi-fluorescence microscope.

Supplementary Figure 2 Correlation between biofilms formed on the polyurethane (B_Rel_) and glass surfaces of the clinical *P. mirabilis* strains.

Supplementary Table 1 Susceptibility of the *P. mirabilisisolates* used in the study.
