# Supplementary material for: Pathogenic Factors Correlate With Antimicrobial Resistance Among Clinical Proteus mirabilis Strains
Source: Front Microbiol. 2020 Nov 25;11:579389. doi: 10.3389/fmicb.2020.579389 (PMC7723865; doi:10.3389/fmicb.2020.579389)
Supplement: Supplementary Table 1 — Susceptibility of the P. mirabilisisolates used in the study. [file Table_1.DOCX]

**TABLE S1**. Susceptibility of the *P. mirabilis* isolates used in the study.

| Isolate | β-lactamases | MICs (mg/L)*^a, b^* | | | | | | | | | | | | | | | | | | |
| --- | --- | --- | --- | --- | --- | --- | --- | --- | --- | --- | --- | --- | --- | --- | --- | --- | --- | --- | --- | --- |
|  |  | AMP | AMC | PIP | TZP | FOX | CAZ | CTX | FEP | ATM | IPM | MEM | GEN | AMK | NET | TOB | CIP | NOR | SXT | CHL |
| 27 | CMY-12+TEM-2-like | **>512** | **64** | **512** | **64** | **256** | **128** | **128** | **8** | **8** | *4* | 2 | **128** | *16* | **64** | **16** | **64** | **>128** | **64** | **128** |
| 305 | CMY-2-like+TEM-1-like | **>512** | **>512** | **256** | *16* | **128** | **32** | **128** | *4* | 0.5 | *4* | 0.125 | 2 | 0.5 | **8** | 1 | **16** | **16** | **32** | nd |
| 777 | CMY-2-like+TEM-2-like | **>512** | **>512** | **256** | *16* | **32** | **16** | **64** | *2* | *2* | *4* | 0.125 | **256** | **>512** | **>512** | **>512** | **32** | **32** | **>64** | **128** |
| 845 | CMY-2-like+TEM-1-like | **>512** | **32** | **256** | 4 | **32** | *4* | *2* | *2* | 0.25 | *2* | 0.125 | **128** | 2 | **32** | **8** | **8** | **16** | **>64** | **>128** |
| 1180 | CMY-2-like+TEM-1-like | **>512** | **64** | **256** | *16* | **128** | **128** | **128** | **8** | **16** | *4* | 0.125 | **128** | 2 | **32** | **8** | **>64** | **>128** | **>64** | **128** |
| 1181 | CMY-2-like+TEM-1-like | **>512** | **64** | **256** | *16* | **128** | **128** | **128** | **8** | **8** | *4* | 0.125 | **64** | 1 | **16** | 4 | **>64** | **>128** | **>64** | **>128** |
| 1376 | CMY-45+TEM-1 | **>512** | **64** | **128** | 4 | **128** | **128** | **64** | *2* | **8** | *4* | 0.06 | 2 | 8 | **64** | **8** | **32** | **128** | **>64** | **>128** |
| 1608 | CMY-15+TEM-1-like | **>512** | **32** | **128** | **32** | **128** | **32** | **128** | *4* | *4* | *2* | 0.06 | **128** | **128** | **64** | **8** | **>64** | **>128** | **>64** | **128** |
| 1671 | CMY-15+TEM-1-like | **>512** | **64** | **512** | **32** | **32** | **32** | **64** | *4* | *2* | *2* | 0.125 | **256** | **64** | **256** | **64** | **64** | **64** | **>64** | **128** |
| 2014 | CMY-15+TEM-1-like | **>512** | **128** | **256** | *16* | **128** | **32** | **128** | *4* | *2* | *4* | 0.25 | **128** | **256** | **64** | **16** | **4** | **16** | 2 | **128** |
| 3010 | CMY-2-like+TEM-1-like | **>512** | **64** | **512** | **128** | **512** | **256** | **128** | **8** | **8** | *4* | 0.25 | **64** | **64** | **16** | 4 | **>64** | **>128** | **64** | **128** |
| 6103 | CMY-15+TEM-2-like | **>512** | **128** | **128** | 8 | **128** | **128** | **64** | *4* | *4* | **8** | 0.5 | **32** | 0.5 | **16** | 4 | **64** | **128** | *4* | **128** |
| 6173 | CMY-2-like+TEM-2-like | **>512** | **64** | **512** | **64** | **256** | **64** | **128** | *4* | **8** | *4* | 0.125 | **128** | 1 | **32** | **16** | **8** | **16** | **8** | **128** |
| 6178 | CMY-2-like+TEM-2-like | **>512** | **64** | **512** | **64** | **256** | **64** | **64** | *2* | *4* | *4* | 0.25 | **128** | **128** | **64** | **8** | **8** | **16** | **>64** | **128** |
| 6184 | CMY-2-like+TEM-2-like | **>512** | **64** | **>512** | **64** | **128** | **64** | **128** | *2* | **8** | *2* | 0.125 | **128** | **256** | **64** | **16** | **8** | **16** | **>64** | **128** |
| 6187 | CMY-2-like+TEM-1-like | **>512** | **64** | **512** | **64** | **256** | **64** | **128** | *4* | *4* | *4* | 0.25 | **128** | **256** | **64** | **8** | **8** | **16** | **>64** | **128** |
| 6199 | CMY-2-like+TEM-2-like | **>512** | **64** | **>512** | **64** | **256** | **64** | **128** | *4* | **8** | *4* | 0.25 | **128** | **256** | **32** | **16** | **8** | **16** | **>64** | **128** |
| 6422 | CMY-12+TEM-2-like | **>512** | **>512** | **128** | **256** | **32** | **32** | **64** | *4* | *2* | *2* | 0.06 | **64** | 0.5 | **64** | **8** | **16** | **4** | **>64** | **>128** |
| 6446 | CMY-2-like+TEM-1-like | **512** | **64** | **512** | **32** | **256** | **64** | **256** | **16** | *4* | *4* | 0.125 | **128** | **>512** | **>512** | **512** | **2** | **4** | **>64** | **128** |
| 6735 | CMY-14+TEM-1-like | **>512** | **64** | **64** | 2 | **32** | **16** | **32** | *4* | 1 | *2* | 0.06 | **256** | 2 | **512** | **64** | **1** | **2** | **>64** | **128** |
| 6769 | CMY-15+TEM-2-like | **>512** | **128** | **>512** | **128** | **64** | **32** | **128** | **8** | *2* | *4* | 0.25 | **>256** | **>512** | **32** | **32** | **>64** | **128** | 2 | **128** |
| 8345 | CMY-12+TEM-2-like | **512** | **64** | **256** | *16* | **32** | **64** | **128** | **8** | *4* | *2* | 0.125 | **128** | **512** | **128** | **16** | **8** | **8** | **>64** | **128** |
| 2094 | CTX-M-1-like+TEM-1-like | **>512** | 2 | **32** | ≤0.25 | 4 | 0.25 | **8** | **8** | 0.125 | *0.5* | 0.03 | **128** | **>512** | **>512** | **512** | **32** | **64** | **>64** | **64** |
| 2029 | TEM-1-like | **>512** | **16** | **128** | 0.5 | 4 | ≤0.03 | ≤0.015 | 0.25 | ≤0.03 | *0.25* | 0.06 | **8** | 0.5 | 2 | 1 | 0.25 | 0.5 | **>64** | **128** |
| 2198 | TEM-1-like | **256** | 2 | 2 | 0.5 | 4 | 0.25 | ≤0.015 | 0.125 | ≤0.03 | *0.25* | 0.06 | **32** | *16* | **256** | **128** | 0.25 | 0.5 | **>64** | **16** |
| 368 | - | ≤1 | ≤1 | ≤0.5 | ≤0.25 | 2 | ≤0.03 | ≤0.015 | ≤0.008 | ≤0.03 | *0.5* | 0.06 | 0.25 | 1 | 0.25 | 0.5 | 0.015 | 0.06 | 0.06 | 4 |
| 5112 | - | ≤1 | ≤1 | ≤0.5 | ≤0.25 | 2 | ≤0.03 | ≤0.015 | 0.06 | ≤0.03 | *1* | 0.06 | 0.25 | 0.5 | 0.5 | 0.25 | 0.03 | 0.06 | ≤0.125 | 4 |
| 5568 | - | ≤1 | ≤1 | ≤0.5 | ≤0.25 | 2 | ≤0.03 | ≤0.015 | 0.125 | ≤0.03 | *1* | 0.06 | 0.25 | 0.5 | 0.5 | 0.25 | 0.06 | 0.125 | 0.06 | 4 |
| 5618 | - | ≤1 | ≤1 | ≤0.5 | ≤0.25 | 4 | ≤0.03 | ≤0.015 | 0.06 | ≤0.03 | *2* | 0.06 | ≤0.125 | 0.5 | 0.25 | 0.25 | 0.03 | 0.06 | ≤0.125 | 4 |
| 5622 | - | ≤1 | ≤1 | ≤0.5 | ≤0.25 | 2 | ≤0.03 | ≤0.015 | 0.125 | ≤0.03 | *1* | 0.06 | 0.25 | 0.5 | 0.5 | 0.25 | 0.06 | 0.06 | ≤0.125 | 4 |
| 5653 | - | ≤1 | ≤1 | ≤0.5 | ≤0.25 | 4 | ≤0.03 | ≤0.015 | 0.125 | ≤0.03 | *1* | 0.06 | 0.25 | 0.5 | 0.5 | 0.5 | 0.03 | 0.06 | 1 | 8 |
| 5663 | - | ≤1 | ≤1 | ≤0.5 | ≤0.25 | 2 | ≤0.03 | ≤0.015 | 0.125 | ≤0.03 | *0.25* | 0.06 | 0.25 | 0.5 | 0.25 | 0.25 | 0.03 | 0.125 | 0.25 | 8 |
| 5777 | - | ≤1 | ≤1 | ≤0.5 | ≤0.25 | 1 | ≤0.03 | ≤0.015 | 0.06 | ≤0.03 | *0.25* | 0.06 | 0.25 | 0.5 | 0.5 | 0.25 | 0.03 | 0.06 | 0.06 | 8 |
| 5778 | - | 2 | ≤1 | ≤0.5 | ≤0.25 | 4 | 0.06 | ≤0.015 | 0.125 | ≤0.03 | *0.25* | 0.06 | 0.5 | 2 | 1 | 0.5 | 0.015 | 0.125 | 0.06 | 8 |
| 6181 | - | ≤1 | ≤1 | ≤0.5 | ≤0.25 | ≤0.5 | 0.06 | 0.06 | ≤0.008 | 0.06 | 0.125 | 0.03 | 0.5 | 0.5 | 0.25 | 0.5 | 0.03 | 0.06 | 1 | 8 |
| 6185 | - | ≤1 | ≤1 | ≤0.5 | ≤0.25 | ≤0.5 | 0.06 | ≤0.015 | 0.015 | ≤0.03 | 0.03 | 0.125 | 0.125 | 0.5 | 0.25 | 0.25 | 0.03 | 0.06 | ≤0.125 | 8 |
| 6365 | - | 2 | ≤1 | ≤0.5 | ≤0.25 | 4 | 0.06 | ≤0.015 | 0.125 | ≤0.03 | *0.25* | 0.03 | 0.25 | 0.5 | 0.25 | 0.25 | 0.015 | 0.06 | ≤0.125 | 8 |
| 6405 | - | ≤1 | ≤1 | ≤0.5 | ≤0.25 | 4 | ≤0.03 | ≤0.015 | 0.06 | ≤0.03 | 0.125 | 0.03 | 0.25 | 0.5 | 0.5 | 0.5 | 0.015 | 0.06 | ≤0.125 | 8 |
| 6519 | - | 2 | ≤1 | ≤0.5 | ≤0.25 | 4 | ≤0.03 | ≤0.015 | 0.125 | ≤0.03 | *1* | 0.06 | 0.25 | 1 | 0.25 | 0.25 | 0.03 | 0.06 | ≤0.125 | 8 |
| 6521 | - | 2 | ≤1 | ≤0.5 | ≤0.25 | 2 | ≤0.03 | ≤0.015 | 0.06 | ≤0.03 | *0.25* | 0.06 | ≤0.125 | 0.5 | 0.25 | 0.125 | 0.015 | 0.06 | ≤0.125 | 8 |
| 6684 | - | ≤1 | ≤1 | ≤0.5 | ≤0.25 | 2 | ≤0.03 | ≤0.015 | 0.06 | ≤0.03 | 0.125 | 0.06 | 0.25 | 0.5 | 0.5 | 0.25 | 0.03 | 0.06 | 0.06 | 4 |
| 6711 | - | ≤1 | ≤1 | ≤0.5 | 0.5 | 2 | ≤0.03 | ≤0.015 | 0.03 | ≤0.03 | 0.125 | 0.06 | ≤0.125 | 0.5 | 0.25 | 0.25 | 0.03 | 0.06 | 0.06 | 8 |
| 6739 | - | ≤1 | ≤1 | ≤0.5 | ≤0.25 | 4 | ≤0.03 | ≤0.015 | 0.06 | ≤0.03 | 0.125 | 0.06 | ≤0.125 | 0.5 | 0.5 | 0.5 | 0.015 | 0.06 | ≤0.125 | 4 |
| 6771 | - | 2 | ≤1 | ≤0.5 | ≤0.25 | 4 | ≤0.03 | ≤0.015 | 0.06 | ≤0.03 | *1* | 0.125 | ≤0.125 | 0.5 | 0.25 | ≤0.125 | 0.03 | 0.06 | ≤0.125 | 8 |
| 7101 | - | ≤1 | ≤1 | ≤0.5 | ≤0.25 | 4 | ≤0.03 | ≤0.015 | 0.125 | ≤0.03 | *0.5* | 0.06 | 0.25 | 2 | 0.25 | 0.125 | 0.03 | 0.06 | ≤0.125 | 8 |
| 7104 | - | ≤1 | ≤1 | ≤0.5 | ≤0.25 | 4 | ≤0.03 | ≤0.015 | 0.06 | ≤0.03 | *0.5* | 0.06 | 2 | 4 | 1 | 0.5 | 0.015 | 0.06 | ≤0.125 | 8 |
| 7491 | - | ≤1 | ≤1 | ≤0.5 | ≤0.25 | 4 | ≤0.03 | ≤0.015 | ≤0.008 | ≤0.03 | *0.25* | 0.06 | 0.5 | 1 | 0.5 | 0.25 | 0.06 | 0.06 | 0.25 | 8 |
| 7498 | - | ≤1 | ≤1 | ≤0.5 | ≤0.25 | 4 | ≤0.03 | ≤0.015 | ≤0.008 | ≤0.03 | *0.5* | 0.06 | ≤0.125 | 0.5 | 0.125 | ≤0.125 | 0.03 | 0.06 | 0.25 | 4 |
| 7499 | - | ≤1 | ≤1 | 1 | ≤0.25 | 2 | ≤0.03 | ≤0.015 | ≤0.008 | ≤0.03 | *0.25* | 0.06 | ≤0.125 | 1 | 0.25 | 0.25 | 0.03 | 0.06 | ≤0.125 | 8 |
| 7882 | - | ≤1 | ≤1 | ≤0.5 | ≤0.25 | 1 | ≤0.03 | 0.015 | 0.06 | ≤0.03 | *1* | 0.03 | ≤0.125 | 0.5 | 0.25 | 0.5 | 0.06 | 0.06 | 0.06 | 4 |

*^a^* – the MICs values shown in bold, italic and normal style correspond to resistance, susceptible – increased exposure and susceptibility, respectively.

*^b^* – abbreviations: AMC; amoxicillin-clavulanate; AMK, amikacin; AMP, ampicillin; ATM, aztreonam; CAZ, ceftazidime; CHL, chloramphenicol; CIP, ciprofloxacin; CTX, cefotaxime; FEP, cefepime; FOX, cefoxitin; GEN, gentamicin; IPM, imipenem; MEM, meropenem; NET, netilmicin; NOR, norfloxacin; PIP, piperacillin; SXT, trimethoprim-sulfamethoxazole; TOB, tobramycin; TZP, piperacillin-tazobactam, nd - not diagnosed
